# A Study on Feto-Maternal Outcome Among Elderly Pregnant Women During the Intrapartum and Postpartum Period in a Tertiary Care Center

**DOI:** 10.7759/cureus.82410

**Published:** 2025-04-16

**Authors:** Amiya Das, Avir Sarkar, Lisley Konar, Bijoya Mukherjee, Rituparna Das, Suman S Samal, Kanishk K Medhavi, Prathamesh Lanjewar

**Affiliations:** 1 Obstetrics and Gynecology, All India Institute of Medical Sciences, Kalyani, Kalyani, IND; 2 Obstetrics and Gynecology, Noida International Institute of Medical Sciences, Noida, IND

**Keywords:** advanced age, antenatal complications, elderly pregnancy, feto-maternal outcome, pregnancy complications

## Abstract

Introduction: The rising incidence of pregnancies among older women, typically defined as those aged 35 and above, has raised concerns regarding both maternal and fetal health outcomes. Advanced maternal age is linked to various pregnancy complications, such as a higher risk of chromosomal abnormalities, gestational hypertension, preterm birth, and other adverse outcomes. This prospective cohort study aimed to investigate maternal and fetal outcomes in this population of elderly pregnant women, shedding light on the risks and complications they face during pregnancy. The study also explored the prevalence of pregnancy-related issues in older women and how these conditions affected both maternal and fetal health.

Materials and methods: This prospective cohort study was conducted over two years at a tertiary care hospital, with ethics approval and written informed consent obtained. It included pregnant women aged ≥35 attending the antenatal clinic and delivering at the hospital. Women with major pre-existing medical conditions (e.g., severe respiratory or cardiac disease, uncontrolled diabetes or hypertension) were excluded. Data were collected through patient interviews and medical record reviews, covering maternal factors (age, parity, socioeconomic status, religion, obstetric history, and comorbidities) and pregnancy complications (e.g., gestational diabetes, preeclampsia, anemia, cardiac issues, and antepartum hemorrhage). Fetal outcomes, such as gestational age, birth weight, Apgar score, congenital anomalies, and mode of delivery, were documented. Participants were followed from the antenatal period through delivery and postpartum discharge.

Results: Out of 23,777 total recruited participants, 311 were elderly mothers (1.3%). The mean age was 36.2±1.7 years, with most women (89.6%) aged 35-40. The majority were Muslim (89.6%) and from a Class IV socioeconomic background (50%). Delayed childbirth was often due to unknown causes (45.3%) or a preference for male children (35.8%). Delayed childbirth in the study was primarily attributed to unknown causes (45.3%) and a preference for male children (35.8%). The “unknown causes” category likely reflects delayed marriage, career, or education pursuits, infertility issues, or broader societal shifts. In contrast, the preference for male children highlights a cultural influence, where sons are providers of financial and social security. This can lead couples to postpone childbirth or continue trying for a male child, thereby increasing maternal age. Most were multigravida (84.9%), with 58.5% having cesarean sections. Common complications included anemia (24.5%), PIH (9.4%), and preterm labor (21.7%). Most births were live (98.1%), with 32.7% requiring NICU admission due to low birth weight or prematurity, and 17.6% neonatal deaths were recorded.

Conclusion: Elderly pregnancies carry a greater risk for both maternal and fetal complications, including gestational hypertension, preeclampsia, gestational diabetes, low birth weight, preterm labor, and an increased likelihood of cesarean delivery. However, with proper prenatal care, regular monitoring, and timely interventions, many of these risks can be effectively managed to enhance both maternal and fetal health. This study emphasizes the need for personalized care for older pregnant women, advocating for early detection, prevention measures, and a collaborative approach to ensure safe pregnancies in this growing population.

## Introduction

The well-being of society is closely linked to the health status and survival of mothers and children. Pregnancy and childbirth are major life events, symbolizing the transition into motherhood, and the health of the mother and fetus are deeply interconnected. Over recent decades, there has been a shift in the demographic patterns regarding the age of marriage and childbirth, which was initially seen in developed countries but is now also becoming more prevalent in developing nations like India. This has led to an increase in the number of pregnancies among older women, and it is projected that by the end of the 20th century, the number of babies born to elderly women will be double compared to the 1980s [1].

Advanced maternal age (AMA) is generally defined as pregnancy occurring at or after the age of 35. This age threshold was first recommended by the International Federation of Gynecology and Obstetrics (FIGO) in 1958 and has since been widely accepted in many countries. A significant number of women are now opting to delay childbirth into their late 30s and even 40s, a trend that has been observed globally, regardless of race or economic status. Factors contributing to this trend include wider educational and career opportunities for women, greater access to contraceptive methods, and changing social dynamics such as increasing divorce rates [2].

Pregnancies in older women carry elevated risks for both the mother and fetus. Maternal complications include chronic hypertension, diabetes, anemia, subfertility, miscarriage, and preterm labor, which may increase the need for cesarean deliveries or instrumental interventions. Studies have shown that healthy older women who receive appropriate pre-pregnancy counseling and perinatal care can achieve outcomes comparable to younger women. Therefore, elderly mothers require modern and specialized care throughout their pregnancy journey, including pre-conceptional, antenatal, intra-natal, and postnatal care in well-equipped institutions. As the number of older pregnant women increases, it is important to focus on this growing demographic group for further research and improved care strategies.

The present study aims to assess the fetal and maternal risks and outcomes associated with pregnancies in elderly women. It also seeks to identify strategies to mitigate these risks and improve maternal and neonatal health outcomes, particularly in settings with limited resources, such as peripheral care centers.

## Materials and methods

It was a prospective cohort study conducted in a tertiary care teaching institute and hospital over two years to assess the sociodemographic characteristics, maternal health outcomes during pregnancy, and neonatal outcomes in women aged 35 years or older, commonly referred to as elderly pregnant women. As AMA is increasingly prevalent, particularly in both developed and developing nations, this study aimed to explore the risks and complications associated with older maternal age, providing insights into the care and management required for this group. The research was conducted over two years, focusing on outcomes that could guide healthcare improvements for elderly pregnant women.

Before initiating the study, ethics approval was granted by the institutional ethics committee (BSMC/Aca/33: 02-01-18), ensuring that the research adhered to ethical standards and protected participants’ rights. Informed written consent was obtained from each participant after explaining the nature of the study, its procedures, and their right to withdraw at any point. By obtaining this consent, the study followed the principles of respect, beneficence, and justice, ensuring that the rights of participants were safeguarded throughout the research process.

To determine the sample size, the study employed the following formula: n=(zl)2n = \left(\frac{z}{l} \right)^2n=(lz​)2, where z represents the Z-score (1.96 for a 95% confidence interval), and l is the margin of error (set at 20%). Using this formula, the initial sample size was calculated to be 96. To account for a 10% non-response rate, the final sample size was adjusted to 106. The study aimed to include all elderly pregnant women, aged 35 years or older, who visited the antenatal outpatient department, regardless of their gestational period, parity, or gravidity. Participants were consecutively recruited until the sample size target was met, ensuring that the study represented a diverse range of elderly pregnancies.

Exclusion criteria were applied to focus the research on the target population. Women who delivered outside the hospital or in the emergency room, those who refused to provide consent, or those with severe co-morbidities were excluded. This exclusion helped avoid potential confounding factors that could affect the outcomes of the study and ensured that the findings were specific to pregnancies in older women.

The data collection process was comprehensive, combining structured interviews, clinical examinations, and a review of relevant medical records. This approach ensured that both maternal and neonatal health outcomes were thoroughly documented.

For maternal data, sociodemographic details such as age, weight, height, body mass index (BMI), socioeconomic status, and religion were recorded, as these factors can influence both pregnancy and health outcomes. Additionally, obstetric history was collected, including information on parity, gravidity, and any previous pregnancies, as these factors may contribute to risks in AMA pregnancies.

The study also examined pre-existing conditions like anemia, diabetes, and hypertension, as these are more prevalent among older pregnant women and can complicate pregnancies. Maternal diseases related to pregnancy, such as gestational hypertension, pre-eclampsia, and gestational diabetes, were monitored as they pose significant risks during pregnancy and delivery, especially for older women. The mode of delivery (vaginal or cesarean) was recorded, as elderly women are more likely to experience complications requiring surgical intervention [3].

Antenatal complications, including preterm labor, malpresentation, and other pregnancy-related issues, were also documented, as they contribute to maternal and fetal health risks. Postpartum complications, such as infections or hemorrhage, were noted to complete the picture of maternal health.

For neonatal data, key parameters such as fetal growth, birth weight, and Apgar scores were measured. Neonatal morbidity, including birth asphyxia, respiratory distress, and other complications, was assessed. The study also examined whether the neonate required resuscitation or admission to the Special Newborn Care Unit (SNCU), as well as the occurrence of fetal or neonatal death. The length of hospital stay for both the mother and neonate was also recorded, as it serves as an indicator of the severity of any complications.

Throughout the study, participants were closely monitored during the intrapartum and postpartum periods, with daily assessments of both the mother and newborn. This regular monitoring allowed for early identification of complications and ensured that the necessary interventions were provided. The collected data were analyzed using Microsoft Excel and SPSS version 20.0 (IBM Corp., Armonk, NY), employing descriptive statistics such as means, standard deviations, medians, frequencies, and percentages. The analysis helped identify patterns and correlations between various sociodemographic factors, maternal conditions, and neonatal outcomes.

## Results

Among 23,777 participants, 311 (1.3%) were elderly mothers, with a mean age of 36.2 years (range 35-42 years). The majority of elderly mothers (89.6%) were in the age group of 35 to less than 40 years. The study showed a predominance of Muslim women among the elderly pregnant population, with 89.6% identifying as Muslim, compared to 42.9% Hindu women. In terms of education, 38.7% of the participants had completed middle school, 29.2% had finished secondary school, and a small portion, 3.8%, were illiterate. Regarding socioeconomic status, half of the participants (50%) belonged to Class IV according to the modified BG Prasad socioeconomic scale (2018).

The main reasons for delayed childbirth were not explicitly known (45.3%) or a preference for a male child (35.8%). A smaller proportion (5.9%) had primary infertility, which was treated with methods such as in vitro fertilization (IVF) or ovulation induction. The majority of the study population (66.04%) delivered at a gestational age between 37 and 40 weeks, while 22.6% delivered prematurely (before 37 weeks).

Regarding BMI, the majority of participants (56.6%) had a BMI between 18.5 and 24.9, indicating a normal weight. Approximately 32.1% were overweight, and the rest had other BMI classifications. A significant number of the participants were multigravida, with 84.9% having been pregnant previously. First-time mothers (primigravida) made up 15.1% of the study population.

Obstetric outcomes

The mode of delivery was varied, with 58.5% of deliveries performed via cesarean section. Normal vaginal delivery accounted for 33.9%, and forceps were used in 3.8% of cases. The majority of the women had a singleton pregnancy (90.5%), while 9.5% had multiple pregnancies. The duration of labor for most women (59.1%) was less than 12 hours.

Medical complications

Several medical complications were observed during the pregnancies. A quarter of the study population (24.5%) had anemia. Other complications included pregnancy-induced hypertension (PIH) in 9.4%, preeclampsia in 7.5%, eclampsia in 3.8%, gestational diabetes mellitus (GDM) in 3.8%, and heart disease in 2.9%. The remaining 49% of the participants had no significant medical complications during pregnancy (Table 1).

**Table 1:** Distribution of participants according to medical complications PIH: Pregnancy-induced hypertension, GDM: Gestational diabetes mellitus

Medical complications	Frequency (n=106), multiple comorbidities present	Percentage
PIH	10	9.4
Preeclampsia	8	7.5
Eclampsia	4	3.8
GDM	4	3.8
Anaemia	26	24.5
Heart disease	3	2.9
Others	14	13.2
Nil	52	49

Obstetric complications

In terms of obstetric complications, preterm labor occurred in 21.7% of cases, while post-dated pregnancies were observed in 18.9%. Premature rupture of membranes (PROM) was seen in 15.1%, and scanty amniotic fluid was noted in 9.4% of pregnancies. Despite these complications, a majority of cases (81.1%) did not require induction of labor. 

Postpartum complications

Postpartum complications were also recorded. Postpartum hemorrhage (PPH) occurred in 18.9% of the cases, followed by abdominal distension in 9.4% and wound infections in 7.5%. However, 61.3% of the participants experienced an uneventful postpartum period.

Neonatal outcomes

The majority of elderly mothers in the study had live births, with 98.1% of babies surviving. However, 1.9% of pregnancies resulted in intrauterine fetal death (IUFD). Regarding birth weight, 39.6% of babies had a birth weight greater than 2.5 kg, while 54.7% had a birth weight ranging from 1.5 to 2.5 kg. A significant proportion of babies (32.7%) required admission to the SNCU.

The most common reasons for SNCU admission were low birth weight (82.3%), prematurity (58.8%), and delayed crying after birth (47.1%). Only a small proportion (5.88%) of babies were admitted due to congenital anomalies. Neonatal deaths occurred in 17.6% of the cases where the baby was admitted to the SNCU.

This study highlights the challenges faced by elderly pregnant women, including a higher prevalence of medical and obstetric complications, as well as increased risks for adverse neonatal outcomes. The data underscores the need for careful monitoring and management of elderly pregnancies to reduce risks for both the mother and the newborn. Despite the higher incidence of complications, many women in this study experienced successful pregnancies, and with appropriate interventions, such as timely delivery and neonatal care, the outcomes for both mothers and babies can be improved.

## Discussion

Chronic conditions, such as hypertension, diabetes mellitus, and obesity, are more prevalent in older women and can exacerbate pregnancy outcomes. Studies show that the prevalence of hypertensive disorders, such as gestational hypertension and preeclampsia, is significantly higher in older pregnant women compared to younger counterparts [4,5]. The risk of developing preeclampsia increases with maternal age, with women over 40 years being at a higher risk [6].

Diabetes mellitus, particularly GDM, also occurs more frequently in older pregnant women. Studies have revealed that women aged 35 and older have a higher likelihood of developing GDM, which increases the risk of macrosomia, birth injuries, and the need for cesarean delivery [7]. These maternal conditions are often associated with increased morbidity during labor and delivery, necessitating close monitoring and appropriate management to mitigate adverse outcomes.

Obesity, another risk factor associated with AMA, contributes to several complications, including gestational diabetes, hypertension, and the need for cesarean delivery [8]. Older women are also more likely to experience anemia and infections during pregnancy, further complicating the management of elderly pregnancies [9].

Obstetric complications are more prevalent in pregnancies occurring at an AMA. These include a higher risk of preterm birth, fetal growth restriction (FGR), and placenta-related disorders. Elderly pregnant women are at an increased risk of delivering preterm infants, which can be attributed to underlying medical conditions such as hypertension, diabetes, and other pregnancy-related complications [10]. Additionally, AMA is associated with a higher incidence of FGR, as aging placentas may function less efficiently, leading to reduced blood flow to the fetus [11].

Another significant risk for elderly mothers is the increased likelihood of multiple gestations, especially with the use of assisted reproductive technologies (ART), which are more common among older women. A study by Hohmann et al. found that the use of ART in women aged 35 years and older significantly increases the chances of multiple pregnancies, which in turn leads to higher rates of preterm birth and low birth weight [12].

Placental complications, such as placenta previa and placental abruption, are also more common in older women. These conditions increase the likelihood of hemorrhage during delivery and may necessitate a cesarean section [13]. Additionally, older women are more likely to experience malpresentation, including breech or transverse lie, which increases the risk of obstructed labor and necessitates intervention during delivery [14].

The mode of delivery in elderly pregnancies tends to differ significantly from younger pregnancies, with a higher incidence of cesarean section. Several studies have reported an increased likelihood of cesarean delivery among women aged 35 years and older, primarily due to obstetric complications such as fetal distress, failure to progress, and malpresentation [15]. The increased incidence of cesarean section is also linked to the higher prevalence of comorbid conditions such as hypertension and diabetes, which may increase the need for surgical intervention.

Elderly pregnancies are associated with a higher risk of adverse fetal and neonatal outcomes. Chromosomal abnormalities, particularly Down syndrome, are more common in pregnancies involving older women. The risk of Down syndrome increases significantly after the age of 35, making prenatal screening and genetic counseling essential for elderly mothers. In addition to chromosomal abnormalities, the likelihood of stillbirth and neonatal death is higher in AMA pregnancies, particularly in the presence of comorbid conditions such as hypertension or diabetes.

FGR is also a common concern in elderly pregnancies. Reduced placental function due to maternal age may result in inadequate fetal growth, which increases the risk of preterm birth, low birth weight, and the need for intensive neonatal care [16]. Increased NICU admissions are another important aspect of neonatal outcomes in elderly pregnancies. Due to the higher incidence of preterm birth, low birth weight, and FGR, neonates born to older mothers are more likely to require intensive care following birth [16]. Additionally, these neonates are at a higher risk of respiratory distress syndrome, jaundice, and other neonatal complications, which further contribute to the need for specialized care.

The study had a few limitations. It was an observational study rather than a randomized controlled trial, which could have provided more robust results if conducted across different parameters. A significant number of patients were referred from rural areas, limiting access to proper antenatal care. No comparative data are available regarding the outcomes of spontaneous conception versus IVF pregnancies. Additionally, follow-up after discharge for both mothers and babies was not possible, preventing the assessment of long-term outcomes. Similarly, after admission to the SNCU, the babies' management and day-to-day progress could not be monitored.

## Conclusions

This study underscores the increased risks associated with AMA, including a higher incidence of medical and obstetric complications, such as anemia, hypertensive disorders, and preterm labor. Neonatal challenges, particularly low birth weight and the need for SNCU admission, were also more common in this group. Despite these concerns, the majority of elderly mothers had successful outcomes with timely medical care. These findings highlight the importance of targeted antenatal monitoring and comprehensive perinatal support to improve maternal and neonatal outcomes in elderly pregnancies.
